# Linking real estate data with entrepreneurial ecosystems: Coworking spaces, funding and founding activity of start-ups

**DOI:** 10.1016/j.dib.2021.107185

**Published:** 2021-05-29

**Authors:** Felix Gauger, Jan-Oliver Strych, Andreas Pfnür

**Affiliations:** a Technical University (TU) of Darmstadt, Germany; b Karlsruhe Institute of Technology (KIT), Germany

**Keywords:** Coworking space, Office market, WeWork, Entrepreneurial clusters, Start-up activity, Funding rounds, Entrepreneurial activity, Regional entrepreneurial cluster, New work

## Abstract

This data article describes a panel dataset that combines flexible office space market data with entrepreneurial data, such as founding and funding of ventures in 47 European cities. One adaption of new ways of working are coworking spaces. They are shared working environments that offer office space and intangible resources, such as knowledge sharing, collaboration and networking. Access to flexible office space for self-employed, start-ups, and corporates is a key resource for businesses. Covid-19 has shown that space provision is becoming more flexible and ventures increasingly use scalable space instead of long-term lease agreements for office space or than owning it. Deskmag counts 18,700 coworking spaces worldwide in the year of 2018 with 1.65 million coworkers and high future growth expectations after COVID-19 [1].

Data were collected through two sources. Data about coworking spaces were collected through a web scraper crawling for coworking spaces within a city as of December 31, 2018. Those data were manually enriched by real estate and economic variables, such as the office high prime rent and office market size. Data about the funding and founding of ventures were obtained through using the database Crunchbase, including all start-ups in a city with their type of funding (including: seed, venture capital, private equity, debt convertibles and others) and their financing rounds. The Crunchbase database lists mostly young firms, commonly called start-ups and small medium enterprises (SME), and their financing with external funding. It includes firms that have needed or might need funding in the near future, or have already got funding. Hence, it is possible to relate spatial clusters with entrepreneurial activity and analyze for example the influence of (flexible) office markets on founding activity.

This dataset enables researchers and practitioners to further explore important questions regarding the nexus between the real estate industry, entrepreneurship behavior, start-ups and regional clusters. Due to the scarcity of publicly available quality flexible office space market data, the dataset detailed in this article may play a relevant role to be ready to be used by researchers and practitioners. Funding data can be used for regional analysis, growth development, or any other economic issues.

## Specifications Table

**Table:** 

Subject	Real Estate Economics
Specific subject area	Flexible Office market and business creation of start-ups.
Type of data	Excel file, Stata file
How data were acquired	Data about coworking spaces were obtained through a publicly available, fee required Google Maps Crawler called Botsol (https://www.botsol.com/). Data about of ventures were obtained through the REST API of Crunchbase.com. A Crunchbase's Academic Research Access is necessary in order to use the API. We then combined these data into one data set.
Data format	Clustered and filtered
Parameters for data collection	Start-up data: Only ventures that were at least 10 years old (definition of start-up) were regarded. Coworking data: Coworking spaces were regarded if these spaces deliver rentable flexible office space. Agencies or platforms were not included. Hybrid spaces that offer flexible office space (such as Regus) were also included in the data.
Description of data collection	We used Botsol Google Maps Crawler to crawl the coworking spaces. We selected the region of interest and added the keyword *coworking* in the Botsol Crawler. Data were saved as .csv document and every coworking spaces was manually checked. We added the year of the foundation, that was found through the website or press release. If the founding year was not found, we used Google Maps Review Crawler from Botsol and crawled the reviews of the coworking space. We then replaced the founding year with the year of the first review. We used the Crunchbase v3.0 API to obtain data about ventures and their funding. To connect to the read-only REST API from Crunchbase, json files were written to download a csv export of the data set containing information about the ventures (year of founding) and their funding (external capital and number of funding rounds) in a region. Information how to use the newer version v4.0 of Crunchbase REST API are available at: https://data.crunchbase.com/docs/using-the-api
Data source location	http://www.Crunchbase.com (granted access to publish aggregate data about ventures). http://www.maps.google.de for crawling data about coworking spaces. Bureau of Statistics: httt://www.statista.de (Statistisches Bundesamt)
Data accessibility	With the article
Related research article	Gauger, F., Pfnür, A., & Strych, J.‑O. (2021). Coworking Spaces and Start-ups: Empirical Evidence from a Product Market Competition and Life Cycle Perspective. Journal of Business Research, 132, 67–78. https://doi.org/10.1016/j.jbusres.2021.04.008

## Value of the Data

• The data is unique in providing panel data of German large coworking space markets regarding their size by aggregating data on inception dates and existence of underlying coworking spaces. So, unobserved heterogeneity of coworking space markets across cities can be accounted for in panel regression models. The data's primary beneficiaries include real estate, entrepreneurship, financial, and regional development scholars to analyse business creation processes, (office) market characteristics, and entrepreneurial clusters.
• Descriptives can be employed to understand geographical start-up clusters, further trends, and anomalies in the data.
• Elaborated experiments with industry effects, types of firms, types of funding (venture capital, seed capital, private equity) can be performed in quasi natural experiments. Cluster analysis, propensity matches or regression analysis can be performed using the data.
• This dataset provides extensive information on the spread of coworking spaces in European cities combined with entrepreneurial activity in that cities ready-to-use.

## 1 Data Description

Flexible office space serves the future of work and is a key determinant in an entrepreneurial ecosystem. COVID-19 has accelerated the demand for shared, flexible office space. Within coworking research, most studies are qualitative, as quantitative data are scarce. Therefore, we hand collect data about coworking spaces to create a panel data set through the big 7 cities of Germany, listing all coworking spaces with their numbers and size and the founding and funding activity. The panel includes the years 2010 to 2018. Table 1 lists the variables as collected in the excel file. For the European cities, we include Wework coworking spaces (number and size) and enrich European cities with the number and size of coworking spaces as of the end of 2018. The 47 European cities are the biggest cities in five European countries with the highest GDP. We also provide an overview with the funding dates of WeWork. WeWork is a commercial real estate company that provides shared workspaces (“coworking spaces”) for start-ups and other corporates and is valued like a tech company (see Table 2). It is the leader in Flexible Office Space, counting around 800 flexible office space locations in more than 118 cities around the world (Status: 2021), whereas most of them are found in the United States [2].

**Table 1:** Variable description.

Variable Label	Variable Description	Unit	Source
Year	Year		own research
Region	City in Europe		own research
WeWorkNum	Number of WeWork Coworking Spaces		own research
WeWorkSpace	Size of the WeWork Coworking Space	square meters	own research
NumberCoworkingSpaces	Number of Coworking Spaces opened		own research
SizeCoworkingSpaces	Size of Coworking Spaces opened	square meters	own research
HighPrimeRentOfficeMarket	High Prime Rent of the Office Market	Euro per square meter	CBRE
OfficeMarketTurnover	Office Market turnover	square meters	CBRE
UnemploymentRate	Unemployment Rate	[%]	Bureau of Statistics
GDPperEmployedCapita	Gross Domestic Product per Employed Capita	EUR	Bureau of Statistics
GDPperCapita	Gross Domestic Product per Capita	EUR	Bureau of Statistics
Tax Power	Tax Power	EUR	Bureau of Statistics
Vacancy Rate	Vacancy Rate of Office Space	[%]	CBRE
NumFoundedFirms	Number of founded firms (often called Start-ups and SME)		Crunchbase
AmountAllFunding	Sum of of external funding amount in that city and year, including seed, venture capital, Private equity, convertible debts and others	USD	Crunchbase
RoundNumSeed	Sum of external funding rounds in that city and year		Crunchbase
AmountSeed	Sum of seed capital in that city and year	USD	Crunchbase
RoundNumVCPE	Sum of external funding rounds for venture capital and private equity in that city and year		Crunchbase
AmountVCPE	Sum of venture capital and private equity capital in that city and year	USD	Crunchbase

**Table 2:** Funding of WeWork during establishment in 2010 and 2018.

Year	WeWorkAmountRaised [Mio. USD]	WeWorkAggregate AmountRaised [Mio. USD]	SumWeWork FundingRounds
2020	1,100	20,645.78	17
2019	8,750	19,545.78	16
2018	4,702	10,795.78	12
2017	4,400	6,093.78	9
2016	690	1,693.78	8
2015	433.93	1,003.78	7
2014	355	569.85	6
2013	190	214.85	5
2012	17	24.85	3
2011	7.85	7.85	2

## 2 Experimental Design, Materials and Methods

Data was obtained from a web crawler, called Botsol. Search terms were “coworking”. Data were then manually cleaned and enriched with the size, and inception date of the coworking space. If the founding date was not available, a second web crawler (Botsol Google Maps Review Crawler) searched for the first Google review entry and was set to that year. We replace the missing founding dates with the date of the first Google Maps Review. Due to the novelty of coworking spaces, we find that Google review entries have commonly been used as a rating mechanism for the last years and deliver high-quality results through a “crowd-based approach” [3].

Data about founding and funding of start-ups were obtained by using the crunchbase API with a research access granted by Crunchbase. We wrote json files to download the firms, funding rounds and amount of funding. Those fundings were then aggregated on the city level. If cities were not clearly assignable, those were assigned manually.

Table 2 contains the funding of WeWork from the establishment in 2010 until 2018. and highlights the growth plans of the flexible office space operator. In March 2021, WeWork announced to go public via the special-purpose acquisition company (SPAC) BowX Acquisition Corp. SPAC valuing it at $9 billion USD [4]. WeWork is mainly Softbank-owned and dominates the coworking sector by leveraging data through various domains in real-estate management, office architecture, planning and facility management [5]. Table 2 lists the valuation and funding history of the firm.

## Ethics Statement

None.

## CRediT Author Statement

**Felix Gauger:** Conceptualization, Writing, Data Collection; **Jan-Oliver Strych:** Data Processing, Writing Review & Editing, Validation; **Andreas Pfnür:** Supervision.

## Declaration of Competing Interest

This research did not receive any specific grant from funding agencies in the public, commercial, or not-for-profit sectors. We thank Crunchbase for granting full access for academic purposes.

The authors declare that they have no known competing financial interests or personal relationships which have or could be perceived to have influenced the work reported in this article.

## Appendix Supplementary materials

Image, application 1
